# Lectures to Patients

**Published:** 1921-04-16

**Authors:** 


### Lectures to Patients.

There is a great future (writes a correspondent) for the general practitioner who shall first have the courage to call his patients together and offer them collectively the advice which they ought to seek singly. At first sight the objections to such a course leap to the eye. If people want advice, it is their duty to visit their medical man and pay for it. Unfortunately for themselves, and for the medical man (though not all medical men realise this as clearly as they should), patients do not seek advice until they have actually become patients, save in the cases of a few hypochondriacs who steal the time which should be devoted to more needy people. The emphasis in recent years on the fact that health is a thing to be preserved rather than regained has led many people to consult doctors about things which would formerly have been allowed to develop, but even those who realise their duty to prevent disease by taking action before it has developed are often restrained by the fact that the average practitioner is a busy man, with many urgent calls which seem to them to demand precedence.

Nevertheless, just as a general always tries to attack the enemy before he has dug himself into the trenches, so the attack on disease ought to be concentrated to a far greater extent on incipient and threatened disease. Army examinations showed the terrible toll on fitness taken by childish aches and pains neglected through years because they never became acute enough to seem worth a visit to the doctor. We have not yet got a generation of parents, even among our educated classes, who are capable of recognising the first symptoms even of the more serious diseases. The teaching given in a few schools in in the right direction, but it is only a first step. Most middle-class parents would be glad of an opportunity of meeting the man they know and trust, and a sufficiently large proportion would be ready to recognise the value of his advice given in this way. If a start were made on the local outbreak of some epidemic or at some season of the year when a special topic has unusual application, their interest would be quickened.

Few parents, for instance, during the recent outbreak of sleeping-sickness, did not feel some alarm, and would not have heartily welcomed such guidance as could not possibly have been given by a busy man to his clients individually. The whole subject would have to be approached most carefully and after due consideration of points of etiquette. Questions and answers, the more informal the better, would probably form the most valuable part of the meeting. Any approach to an attempt to turn the meeting into a clinic would have to be resisted. The organisation might be in the hands of a small committee of the medical man's clients. There are great possibilities in the programme for such a series of talks. The doctor ought not to be the loser by lending his support to such an innovation, but there ought to be a double gain. If such a scheme could be tried it would open the way to a new and better relationship between the medical man and his public.

